# Microbiota in Health and Disease—Potential Clinical Applications

**DOI:** 10.3390/nu13113866

**Published:** 2021-10-29

**Authors:** Matthias Laudes, Corinna Geisler, Nathalie Rohmann, Jildau Bouwman, Tobias Pischon, Kristina Schlicht

**Affiliations:** 1 Institute of Diabetes and Clinical Metabolic Research, University Hospital of Schleswig-Holstein, 24105 Kiel, Germany; corinna.geisler@uksh.de (C.G.); nathalie.rohmann@uksh.de (N.R.); kristina.schlicht@uksh.de (K.S.); 2 Division of Endocrinology, Diabetes and Clinical Nutrition, Department of Medicine 1, University Hospital of Schleswig-Holstein, 24105 Kiel, Germany; 3 Microbiology and Systems Biology Group, TNO, Utrechtseweg 48, 3704 HE Zeist, The Netherlands; jildau.bouwman@tno.nl; 4 Molecular Epidemiology Research Group, Max Delbrück Center for Molecular Medicine in the Helmholtz Association (MDC), 13125 Berlin, Germany; tobias.pischon@mdc-berlin.de

**Keywords:** microbiome, gastroenterology, oncology, neurology

## Abstract

Within the last two decades tremendous efforts in biomedicine have been undertaken to understand the interplay of commensal bacteria living in and on our human body with our own human physiology. It became clear that (1) a high diversity especially of the microbial communities in the gut are important to preserve health and that (2) certain bacteria via nutrition-microbe-host metabolic axes are beneficially affecting various functions of the host, including metabolic control, energy balance and immune function. While a large set of evidence indicate a special role for small chain fatty acids (SCFA) in that context, recently also metabolites of amino acids (e.g., tryptophan and arginine) moved into scientific attention. Of interest, microbiome alterations are not only important in nutrition associated diseases like obesity and diabetes, but also in many chronic inflammatory, oncological and neurological abnormalities. From a clinician’s point of view, it should be mentioned, that the microbiome is not only interesting to develop novel therapies, but also as a modifiable factor to improve efficiency of modern pharmaceutics, e.g., immune-therapeutics in oncology. However, so far, most data rely on animal experiments or human association studies, whereas controlled clinical intervention studies are spare. Hence, the translation of the knowledge of the last decades into clinical routine will be the challenge of microbiome based biomedical research for the next years. This review aims to provide examples for future clinical applications in various entities and to suggest bacterial species and/or microbial effector molecules as potential targets for intervention studies.

## 1. Introduction

Microbiome research is a growing field in molecular and clinical nutritional sciences as well as in many different medical disciplines. The reason for the dramatic increase in scientific knowledge in recent years is due to technical advances: while in the past research into gut microorganisms was mainly based on cultivation procedures, presently high throughput genetic sequencing technologies are available [1]. This offers the possibility to examine the microbiome as a whole not relying on species that can be cultured, which is especially of advantage in the gut, where most bacteria are anaerobic and therefore difficult to cultivate. 

Gut microbes have been also of interest as a therapeutic target for decades. Over a century ago, Professor Elie Metchnikoff (*1845-†1916) theorized that health could be enhanced by changing the gut microbiome using host-friendly bacteria found in yogurt [2]. Presently, different pre- and probiotics are on the market as over the counter (OTC) agents for both, prevention and treatment of human diseases. However, the scientific evidence is weak and several of these treatments have not been tested in randomized controlled trials (RCT). In addition, until now the nutrition-host-microbiome interaction is not understood in detail, which is a prerequisite for the development of efficient, targeted and disease-specific microbiome interventions in the future.

In this narrative review we aim to present examples on the potential future role of microbiome-based interventions in different fields of clinical nutrition and medicine, either as an independent therapy or as an adjunct treatment option supporting the effectivity of disease specific pharmacological agents. We will focus on chronic disease states in gastroenterology, oncology, neurology and metabolic medicine, since keyword searches in the Pubmed database revealed convincing evidence from animal models that these entities are most suitable for targeted microbiome-based interventions.

## 2. Targeting the Gut Microbiome in Gastroenterology

Inflammatory bowel disease (IBD) is a chronic inflammatory pathology mainly in the gastro-intestinal tract (GIT) but also with extra-intestinal manifestations comprising Crohn’s disease (CD) and Ulcerative Colitis (UC). While UC involves mainly the colon, CD may affect the whole GIT in form of skipped lesions of inflammation [3]. IBD is a global disease with increasing prevalence, whereby the numbers of patients in Europe account to 2.2 million, presently [4]. While the exact etiopathogenesis is neither known for CD, nor for UC, it is generally accepted that a complex interplay of environmental and genetic factors is important for disease manifestation and progression. Of interest, in children with CD affection, exclusive enteral formula nutrition (EEN) is effective in inducing clinical remission, indicating a major role of the nutrition-microbiome-host axis in IBD [5]. Besides steroids and disease modifying drugs (e.g., azathioprine and biologicals) several studies have been carried out in animal models and humans exploring the role of pre- and probiotics in IBD. Evidence exists for E. coli Nissle 1917 (EcN) especially for UC: in a direct comparison this bacterial strain induced remission rates comparable to mesalazin (68% versus 75%, *p* = 0.0508) with no serious adverse events [6]. While promising results were found also in comparison to 5-Aminosalicylic Acid (5-ASA) [7], one study found significantly lower remission rates comparing EcN to placebo [8].

Another promising prebiotic is VSL#3. While a mouse study showed effects of VSL#3 on inflammation and balance of fecal and mucosal intestinal microbiota in UC-associated carcinogenesis [9], various clinical studies that have been summarized in a systematic review also including meta-analysis, revealed efficacy of VSL#3 in the treatment of active UC both for achievement of clinical remission (OR = 2.40, 95% CI = 1.49–3.88) and clinical responsiveness (OR = 3.09, 95% CI = 1.53–6.25) [10].

From our point of view, the most convincing data for a benefit of probiotics (e.g., De Simone Formulation) exists for the treatment of a pouchitis after proctocolectomy in UC, where remission rates of 16/23 (*p* < 0.01) were reported [11]. In contrast to UC, a recent meta-analysis including 22 studies showed no significant effects of probiotics for induction of remission, maintenance therapy or post-operative relapse in CD [12].

Besides probiotics, fecal microbiota transplantation (FMT) has been examined in IBD to beneficially modify the gut microbiome in affected patients. While the first case report was published as early as in 1989 [13], the efficiency and safety of FMT in UC is still controversial. In a recent meta-analysis including 5 prospective RCT including 292 participants in total the authors described a failed combined endpoint of clinical remission and endoscopic response in 71.4% of the FMT group but 91% in the control group, resulting in a RR of 0.79 (95% CI 0.70–0.88, *p* < 0.0001) [14]. From a safety perspective, 6.8% of the FMT group and 4.8% of the control group reported serious adverse events (SAEs) which was not significantly different. SAEs comprised, among others, worsening IBD manifestation and pneumonia [14]. Similarly, certain beneficial effects of FMT have been reported in Crohn’s disease patients. In a pilot randomized controlled study, Sokol et al. observed steroid free remission rates after 10 and 24 weeks post-procedure of 44.4% (4/9) and 33.3% (3/9) in the sham transplantation group and 87.5% (7/8) and 50.0% (4/8) in the FMT group [15]. In summary, these data indicate that FMT microbiome intervention might be a promising in the short term of IBD treatment. However, as Tan et al. stated before, a “one size fits all” approach is not to be recommended, since host-centric factors like disease progression, genotype and use of medication lead to widely differing treatment success [16].

In the field of gastroenterology, most convincing evidence exists for a microbiome therapy for enterocolitis due to Clostridium difficile (C. diff.) infection (CDI), with response rates around 90% in several independent trials [17]. Even in a recent meta-analysis of FMT in severe and fulminant CDI (including 16 studies representing 676 patients) cure rates of 61.3% were reported [18]. Of interest in that context multiple repeated procedures achieved higher cure rates compared to single FMT (100% versus 75%, *p* = 0.01). 

In contrast to acute Clostridium difficile colitis, FMT is not a suitable method for the treatment of chronic GI diseases (e.g., IBD) due to the need of repetitive treatments. Hence, in order to identify targets for future microbiome therapies for IBD scientist became interested in functional bacterial metabolites which enter the human host and induce local and/or systemic effects on the metabolic and immune systems. In that respect, especially small chain fatty acids (SCFAs) derived from dietary fibre and produced by bacterial fermentation are of interest, since these molecules not only serve as energy supply for colonocytes but also exhibit several effects on the mucosa barrier and on the human physiology [19]. Among SCFA, acetate, propionate and butyrate are thought to be most important [20]. Propionate and butyrate production are more conserved and specific, while the ability of acetate production is spread among bacterial classes [21]. Thus, acetate is found in highest concentrations in the human intestinal lumen [22]. SCFA are absorbed by the colonic mucosa by both, active and passive transport [19]. Noteworthy, only 8–12% of the absorbed butyrate and propionate are found in the blood of the portal vein, whereas most of them are metabolised in colonocytes as energy supply [20].

SCFA are able to influence the gastrointestinal immune system on several levels: Within the intestinal lumen, SCFA are essential in regulating the luminal pH, which vice versa affects the overall composition of the microbiome [23]. In colonocytes, butyrate can induce anti-inflammatory IL10 production by binding to the GPR109A receptor [24]. Butyrate also improves intestinal barrier function e.g., due to stabilizing the transcription factor hypoxia inducible factor (HIF) and induction of expression of genes encoding for tight junction proteins [20]. The cells of the innate immune system in the colonic mucosa are also affected by SCFA. Neutrophiles, for example, produce less TNF-α in response to LPS when incubate with SCFA [25]. In addition, activation of the SCFA receptor GPR109A on macrophages is crucial for the equilibrium of pro- versus anti-inflammatory T-cells, indicating that SCFA also affect the adaptive immune system [26]. From a clinical point of view, these findings are of great importance since SCFA producing bacteria are commonly found reduced in IBD (in both, CD and UC), whereby the most repetitive finding is the reduction of Faecalibacterium prausnitzii in active IBD [19]. In addition, reduction in fecal SCFA concentrations have been associated with disease activity as patients in remission have higher levels of butyrate than patients with severe active disease [27]. Therapeutically, butyrate producing bacteria may be supplemented as targeted probiotics, e.g., Butyrococcus pullicaecorum or high fermentable dietary fibres as prebiotics [28]. In addition, tributyrin may serve as a prodrug to increase butyrate levels when administered orally [29]. Simple embedding SCFA into regular food products will rather not be possible, since especially butyrate has a negative taste and smell. However, a targeted colonic delivery obtained by a slow-release formulation as a “postbiotic” might be a suitable approach [29]. 

While several groups focus on SCFA as microbial effector molecules in IBD, in the last ten years our department has produced convincing evidence for a role of amino acids in this context. Especially tryptophan and its related effector molecules nicotinamide and nicotinic acid are promising candidates for the development of targeted microbiome interventions for IBD [30]. Tryptophan not only serves as a nutritive component but is also involved in the regulation of the innate immune system. Removing tryptophan from the diet increases susceptibility to develop colitis in mice which can be reversed by administration of tryptophan or its metabolite nicotinamide [31]. In humans, serum levels of tryptophan are lower in patients with IBD than healthy controls with stronger reduction in CD compared to UC. Metabolomics analysis in patients revealed activation of the kynurenine pathway, known to be associated with a pro-inflammatory state [30]. Of interest, the composition of the gut microbiome was significantly associated with serum levels of tryptophan clearly indicating a role for gut microbes in that context. Presently a clinical trial for targeting the tryptophan pathway in IBD is undertaken in our department.

## 3. Targeting the Gut Microbiome in Oncology

While the gastroenterological diseases reported so far share a primary chronic inflammatory pathology, microbiome-based research has also been conducted in patients with various forms of gastrointestinal (GI) cancer. Differences in the composition of the gut microbiome were found for gastric and colorectal cancer (CRC), whereby *C. diff.* was found to be enriched to approx. 20% in the latter [32]. Presently, it is thought that unfavourable gut microbiota may promote cancer development by multiple mechanisms, e.g., immune response changes and carcinogenic metabolite production [33]. 

In case of gastric cancer it has been shown that *Helicobacter pylori* is able to induce DNA-damage via Cytotoxin-associated gene A (CagA) together with host-mediated reactive oxygen species (ROS), inflammatory factors and growth factors [33]. In addition, Liang et al. in 2019 found *Shigella* spp. enriched in patients with gastric cancer compared to healthy controls [34]. This gram^-^ bacillus can break through the epithelial barrier and is able to inject plasmid antigens (IpaB, IpaC, IpaD) in macrophages and thereby disintegrate the local immune system. From our point of view the most interesting findings have been reported on *Lactobacillus* spp. While these bacteria are supposed to promote health, several independent groups found *Lactobacillus* abundance increased in gastric cancer whereby one group even suggested to use these bacteria to clearly distinguish patients with gastric cancer from healthy individuals [35]. Lactobacillus mainly increase N-nitroso compounds and activate ROS which may result in increased epithelial-mesenchymal transition of *Helicobacter pylori* with the negative consequences described above [36]. 

CRC is the third most diagnosed cancer in the world, with 1.8 million new cases in 2018 [37]. Although age is a risk factor for CRC, more recently the incidence rate under the age of 50 has increased, clearly indicating importance of dietary and lifestyle factors in relation to genetic predispositions, amongst others via unfavourable gut microbiota [38]. Gut bacteria that have been implicated in CRC are *Fusobacterium nucleatum*, *Enterococcus faecalis*, *enterotoxogenic Bacteroides fragilis*, *Streptococcus gallolyticus* and *Porphyromonas species* [37]. A key pathogenic factor is the loss of the protective colonic mucosal barrier resulting in a greater bacterial translocation and entry of toxic microbial products across the epithelium. These exposures may result in ROS production and release, known to promote neoplastic processes [39]. In addition, loss of beneficial microbiota due to an unhealthy diet may result in decreasing SCFA supply, which are involved in maintaining the mucosal barrier function [40]. Beside influencing the barrier function, certain gut bacteria factors may directly induce pro-oncogenic properties by interacting with the 7α-hydroxyl group in specific bile acids inducing cytotoxic 7α-dehydroxylation which is linked to colonic adenoma growth [41]. 

From a clinician’s point of view not only the potential role of the gut bacteria in the induction and progression of GI tumors are of interest, but also the effects of the microbiome on the efficiency of anti-tumor therapies [42] as well as on negative side effects, e.g., fatigue [43]. The activation of the host immune system to defend cancer cells is particularly interesting in modern oncology and immune checkpoint inhibitors (ICI) have become important in routine clinical care. Examples are anti-programmed death-1 (anti-PD-1) and anti-cytotoxic T-lymphocyte antigen-4 (anti-CTLA-4)-antibodies which have been demonstrated efficient in a wide spectrum of malignancies [44]. Noteworthy, the activity of these therapeutics is affected by the immunomodulatory properties of the gut microbiome by influencing local and systemic immune responses at the level of both, the innate and adaptive immune system. For example, patients who have higher abundances in *Firmicutes* and *Verrucomicrobiota* exhibit a better response to ICI therapy, whereas these enriched in *Proteobacteria* presented an unfavourable outcome [42]. While an antibiotic use especially within two months prior to ICI initiation was associated with lower ICI efficiency [45], a plant-based diet was associated with induction of an “ICI-favouring” gut microbiome [42]. In a randomized clinical trial Davar et al. examined the effects of FMT on anti-pD1 resistance in patients with PD-1- refractory melanoma. FMT lead to clinical benefit in 6 out of 15 cases and also resulted in rapid and durable microbiota perturbation [46]. However, in a systematic literature review from March 2021, Huang et al. were not able to extract a significant number of clinical dietary intervention studies in the context of ICI-therapy, clearly indicating an unmet need for nutrition science in this context [42]. *Akkermansia muciniphila* abundance has been associated with both, a favourable metabolic phenotype (e.g., improved insulin sensitivity, see below) and better therapeutic response to ICI therapy [47]. Several animal studies have demonstrated the beneficial effects of FMT from ICI responders to non-responders and have linked these effects to *Akkermansia muciniphila* [48,49]. The abundance of this bacterium can be increased by caloric restriction and by supplementation with functional food products such as resveratrol and inulin [50]. Hence, a dietary intervention during ICI therapy with such a regime might be an interesting future strategy for a targeted nutrition to benefit cancer treatment. 

Recent research in nutrition and cancer identified an “obesity paradox”: obesity dramatically affects the gut microbiome and directly impacts on cancer promotion in several tissues, but at the same time, patients with obesity benefit stronger from ICI therapy when compared to lean cancer patients [51]. For example, in a clinical ICI study including 2046 patients with metastatic melanoma, obese subjects showed a significant advantage in overall survival with a hazard ratio of 0.64 (95% CI 0.47–0.86) with no difference in adverse events [52]. It should be mentioned that obesity is defined by BMI and BMI does not distinguish between visceral and subcutaneous fat accumulation, hepatic fat content or physical fitness of the individuals, which from our point of view needs to be stratified in future analyses.

The gut microbiome is not only important in respect to cancer development and ICI therapy response, but also for patient’s individual sensation of the disease. Chronic fatigue, for example, is often experienced and significantly affects everyday life. In a recent study including 88 patients with advanced, metastatic, unresectable cancers being in the washout period of a chemotherapy a significant correlation of a specified fatigue score was found with specific gut microbiome changes clearly indicating that cancer patients in the future will benefit from a targeted adjuvant microbiome intervention on several levels [43]. 

## 4. Targeting the Gut Microbiome in Neurology

It is known for many years that patients suffering from Parkinson’s diseases (PD) develop gastrointestinal symptoms and some authors even suggest that this common neurodegenerative disease originates in the gut [53]. Indeed, while PD is mainly defined by motor dysfunction (tremor, rigidity, bradykinesia), GI symptoms like dyspepsia, hypersalivation and constipation usually proceed development of other symptoms [54]. Especially constipation is often accompanied by intestinal inflammation and occurs years before onset of extrapyramidal dysfunction [55]. The pathology of PD is primarily found in the substantia nigra in the brain, showing loss of dopaminergic neurons. Histologically, cytoplasmatic aggregates of abnormal α-synuclein (αSyn) proteins, known as Lewy bodies, are found [56]. In 2008, Del Tredici and Braak for the first time hypothesized that abnormal αSyn originates from the gut and spreads to the brain via the vagus nerve in a “prion-like” manner [57]. αSyn early appears in cells of the enteric nervous system (ENS), both, the glossopharyngeal nerve as well as the vagus nerve [58]. Of interest, the risk for PD was shown to be reduced after vagus nerve amputation as gastric ulcer therapy [59]. Additionally, various animal studies support the existence of a gut-brain-axis in the pathophysiology of PD [60]. 

Several studies reported dysbiosis of the gut microbiome in PD patients. As for IBD patients, butyrate-producing bacteria were reported less abundant in fecal samples of PD patients compared to healthy controls [61]. Convincing evidence from human studies indicates reduced *Prevotellaceae* abundance in PD patients, and *Prevotella* populations have been shown to significantly correlate to PD disease activity scores [62]. In contrast, *Enterobacteriaceae* were found more abundant in PD patients and are differentially linked to subtypes of the disease [63]. 

Besides SCFA, which have been shown to act as beneficial effector molecules also in neuroscience [64], in PD a special microbial metabolite recently came to attention: Amyloid Protein Curli. Curli fibres are a class of functional amyloid fibres being produced amongst others by *Escherichia coli* (*E. coli*). They serve as scaffolds for the biofilm in the extracellular matrix (ECM) and promote both, bacterial adhesion and colonization [65]. However, curli fibres can also serve as Pathogen-Associated Molecular Patterns (PAMP) to trigger systemic inflammation, e.g., via Toll like receptor (TLR)-2 activation of macrophages [66]. In addition, Curli subunits are able to trigger PD progression as shown in animal experiments, in which repeated administration of Curli-producing bacteria resulted in accumulation of intestinal αSyn, astrogliosis and microgliosis in the brain [67].

Giving the convincing evidence of the role of the gut microbiome in PD development and progression, clinical intervention trials with Pro-/Prebiotics and FMT have been performed. Tan et al. recently found an improvement in constipation, stool consistency and quality of life in 34 PD patients treated 4 weeks with multi-strain probiotic capsules compared to 38 placebo treated controls [68]. From a mechanistic point of view, the probiotic strain PXN21 of Bacillus subtilis inhibited αSyn aggregation in a *Caenorhabditis elegans* model of synucleinopathy [69]. In respect to FMT in PD patients, so far only case reports have been published with promising results [70]. These case findings are supported by a report on a PD animal model, where FMT was able to reduce microglia and astroglia activation and increased dopaminergic neurons in the striatum [71]. 

While neurodegenerative diseases like PD were initially in the focus of microbiome research in neuroscience, accumulating evidence also suggest a role for the gut microbiome in chronic neuroinflammation, esp. Multiple Sclerosis (MS). While MS is an autoimmune disease of the central nervous system, the activation of the immune cells first occurs in the periphery, before they enter the brain to induce myelin degeneration and axonal loss [72]. The MS pathology comprises increased numbers of T-Helper-(Th)17 and Th1 lymphocytes while regulatory T-lymphocytes (Treg’s) are functionally impaired [73]. First evidence that the gut microbiome exerts an important role in MS development came from animal studies, in which germ-free mice where protected to develop Autoimmune Encephalomyelitis (EAE), an experimental version of MS in model organisms [74]. In humans, the overall gut microbiome diversity in MS patients was found comparable to healthy controls [75], which is somewhat interesting, since many diseases in different tissue/organs share a reduction in diversity as a common principal. However, on species level alterations in MS patients could be observed, e.g., enrichment of *Methanobrevibacter* and, of interest, *Akkermansia* [76]. As for many other diseases, SCFA display beneficial effects in MS. For example, treatment with butyrate and propionate increased the differentiation of Treg’s [77] and improved integrity of the blood-brain-barrier (BBB) [78]. Of interest, tryptophan metabolites are also of importance in MS, since they have recently been shown to reduce pro-inflammatory Th17 lymphocytes and ameliorate EAE in mice [79,80]. 

Dietary intervention seems efficient as an adjuvant anti-inflammatory treatment especially in MS with a special focus on salt ingestion. A high-salt intake was recently found associated with a reduction of Lactobacillus, a genus beneficially affecting neuro-inflammation [81]. Animal data suggest that this effect is mediated via high-salt induced Th17 differentiation, presumably promoted by bacterial tryptophan metabolites [80]. Promising data were also generated in a study using a probiotic cocktail of eight bacteria, including *Lactobacillus*, in which changes in gut microbiota composition and anti-inflammatory immune responses were found [82]. However, at least from our point of view most convincing data were generated with the SCFA propionate as an adjuvant in MS treatment. In a proof-of-concept study, propionate was given as an add-on to their immunotherapy for two weeks in treatment naïve MS patients. The propionate intake resulted in a significant decrease of Th17 and Th1 cells, while Treg’s significantly increased. Interestingly, the propionate effect was linked to the gut microbiota composition [77]. 

## 5. Targeting the Gut Microbiome in Metabolic Medicine

Obesity, type 2 diabetes and metabolic fatty liver Diseases (MeFLD) are the most prevalent nutrition associated diseases in the western world [83,84]. In the situation of an increasing energy supply accompanied by reduced physical activity, adipose tissue exceeds its ability to store the excess energy as triglycerides resulting in so called ectopic lipid accumulation in metabolic active tissues, e.g., skeletal muscle and liver. Ectopic lipid accumulation interferes with insulin signaling cascades and thereby induces insulin resistance [85]. However, not all insulin resistant subjects develop type 2 diabetes, suggesting additional pathogenic mechanisms. In this respect, besides host genetic factors (e.g., TCFL7 SNP), also the gut microbiome came into the focus of biomedical research [86]. 

Western-diet induced obesity alters the gut microbiome in several ways, e.g., on phylum levels increases the abundance of Firmicutes at the expenses of Bacteriodetes [87]. Also, on species levels significant alterations in obesity have been reported, including reduced abundance in *Akkermansia*, *Faecalibacterium*, *Oscillibacter*, and *Alistipes* [86]. The microbiome can affect human physiology on several ways: (1) several microbes are able to pre-digest otherwise indigestible food components, e.g., fibres into SCFA, which than can be absorbed into the human organism to increase energy supply (=energy harvest theory) [88], (2) microbes can induce a low-grade systemic inflammation e.g., by LPS production or disturbing intestinal barrier (=inflammatory theory) [89], and (3) gut microbes can produce bioactive signaling molecules which regulate/dysregulate human metabolism (=nutrition-microbiome-host metabolic axis) [90]. Especially the latter is currently examined deeply by combining shotgun metagenomic sequencing with untargeted HPLC or NMR metabolomics to identify novel compounds for obesity and diabetes treatment.

Bile acids are among the main metabolites produced/modified by the gut microbiome which are not only important for fat resorption but also for regulating liver metabolic function [91]. The secondary bile acids deoxycholic acid (DCA) and lithocholic acid (LCA), for example, are able to activate the intestinal FXR receptor resulting in the expression of fibroblast growth factor 15 (FGF-15). FGF-15 regulates bile acid hepatic synthesis by reduction of cholesterol 7α-hydroxylase and hepatic glucose metabolism by inhibiting the CREB-PGC-1α pathway [92]. Of interest, ablation of the gut microbiome in a hamster model alleviated high fat induced glucose intolerance, hepatic steatosis and inflammation, indicating the importance of the microbiome-bile acid FXR axis in obesity [93]. 

Besides bile acids, SCFA have also been implicated in obesity and type 2 diabetes development. Butyrate and propionate especially can trigger Glucagon like peptide (GLP)-1 and peptide YY (PYY) thereby affecting satiety feeling [94,95]. In addition, propionate has been shown to induce the anorexigenic hormone leptin in human adipose tissue and SCFAs can also suppress appetite through direct effects in the CNS via induction of GABA neuroglial cycles [96]. From a clinician’s point of view, a human association study comprising 952 normoglycemic individuals found that increased intestinal butyrate production was associated with improved insulin response in an oral glucose tolerance test [97]. Of interest, gut microbes are not only able to produce small fatty acids, but also complex polyunsaturated once (PUFA) via the metabolite 10-hydroxy-cis-12-octadecenoic acid (HYA), which has been shown to induce host resistance to HFD-induced obesity [98].

In Metabolic Steatosis hepatitis (MeSH), the severe from of the MeFLD, increased abundance for *Proteobacteria* and *Enetrobacteriaceae* have been reported [99]. These microbes produce Ethanol by saccharolytic fermentation, which might be key in the transformation from MeFLD into MeSH [99]. Hence, a MeSH is not really alcohol independent, supporting the suggestion not longer to use the older term NASH=non-alcoholic steatosis hepatis.

Bacterial molecules not only affect the host metabolism, but also the host immune system. For example, the microbial compounds taurine, histamine, and spermine modulate the NLRP6 inflammasome signaling, epithelial IL-18 secretion, and antimicrobial (AMP) profiles [90]. Release of LPS from the bacterial wall might activate the host innate immunity via toll-like-receptor activation [89]. Furthermore, certain gut microbes can metabolize dietary tryptophan and generate indol-3-aldehype, which can activate the aryl hydrocarbon receptor (AHR). AHR is a ligand-inducible transcription factor regulating gene expression in immune cells resulting e.g., in IL-22 production in innate lymphoid cells (ILC) in the intestinal mucosa [100]. 

In daily clinical routine, most diabetologists already use microbiome related treatments, even though most clinicians might not realize. It is known for several years that Metformin, the first line antidiabetic agent used in millions of type 2 diabetes every day, may induce intestinal site effects, especially diarrhea, in a significant number of patients. While this was seen as a negative effect for decades, in 2019 it has been shown that besides AMPK activation in the liver, part of the desired pharmacological Metformin action is mediated via the gut microbiome [101]. In this study, Pryor et al. found that Metformin activates certain gut microbes to produce Agmatine by decarboxylation of the food-derived amino acid Arginine which is absorbed into the systemic circulation to exert metabolic effects in humans. Besides Metformin, also Acarbose, an anti-diabetic drug shown to be active also in prediabetes, acts in part via the microbiome. Acarbose modifies the composition of the microbiome thereby altering bile composition and influencing the outcome of type 2 diabetic patients [102]. At an experimental level, our own group developed a controlled iliac release of nicotinic acid (CIR-NA), which serves as a bacterial substrate for NAD^+^ production, inducing significant microbiome changes towards a non-diabetic composition and at the same time improves markers of systemic insulin resistance [103]. Presently, a phase 2 clinical trial is performed in a multicentre design in Germany to examine effects of CIR-NA in the treatment of prediabetes to inhibit transition into manifest type 2 diabetes. 

## 6. Summary and Outlook

**Figure 1:**
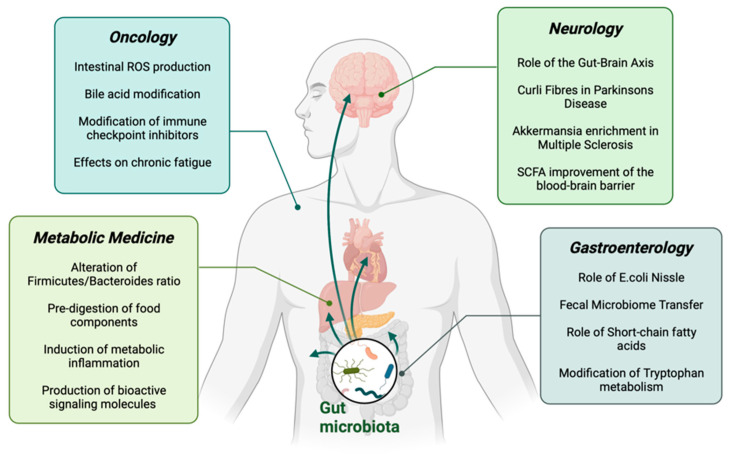
Schematic overview of the main topics in this review. SCFA: Short-Chain Fatty acids.

Figure 1 provides an overview of the topics discussed in this review. 

While this review article cannot cover all clinical aspects of current microbiome research, it is almost certain that targeted interventions will gain future clinical significance in the treatment especially of chronic diseases in various disciplines. Thereby, microbiome therapeutics may either serve as a treatment on its own for the diseases or act as an adjuvant improving the efficiency of a guideline pharmacological agent, as shown for the ICI. The identification of functional active bacterial metabolites (e.g., SCFA or agmatine) by modern metabolomics technologies will enable clinicians in the future not only to use the bacteria itself (e.g., as advanced pre- or probiotics), but also purified or industrial produced molecules as therapeutics (so called post-biotics). In addition, the development of GMP delivery systems to transport microbes and/or metabolites into the terminal ileum or the colon (e.g., CIR-NA), will enable clinicians to alter the microbiome where it is localized, avoiding degradation or modification in the upper intestinal tract. However, it also became clear during the last 10 years that specific interventions will have to be designed for specific diseases since the microbiome is altered differentially in different disease conditions, e.g., CD and type 2 diabetes. While the data so far are very promising, further studies especially in humans will be of importance to finally translate targeted microbiome therapies into clinical routine.

## Data Availability

We exclude this statement.
